# Parallel stent retriever mechanical thrombectomy of an acute internal carotid artery occlusion refractory to standard techniques: A case report

**DOI:** 10.3389/fstro.2023.1066491

**Published:** 2023-02-09

**Authors:** Takeshi Yoshimoto, Satoshi Hosoki, Kanta Tanaka, Junpei Koge, Tetsu Satow, Hiroshi Yamagami, Kazunori Toyoda, Masafumi Ihara

**Affiliations:** ^1^Department of Neurology, National Cerebral and Cardiovascular Center, Suita, Japan; ^2^Division of Stroke Care Unit, National Cerebral and Cardiovascular Center, Suita, Japan; ^3^Department of Cerebrovascular Medicine, National Cerebral and Cardiovascular Center, Suita, Japan; ^4^Department of Neurosurgery, National Cerebral and Cardiovascular Center, Suita, Japan; ^5^Department of Stroke Neurology, National Hospital Organization Osaka National Hospital, Osaka, Japan

**Keywords:** parallel stent retriever technique, mechanical thrombectomy, rescue technique, refractory, standard techniques

## Abstract

Although mechanical thrombectomy for acute large vessel occlusion is generally effective, some occlusions are refractory. We report a patient in whom the parallel stent retriever technique using two Trevo stent retrievers (Stryker Neurovascular, Fremont, California, USA) was required to treat an intracranial and epidural internal carotid artery occlusion after other techniques had failed. A 68-year-old woman presented with an acute left internal carotid artery occlusion 4 days after mechanical thrombectomy of a left middle cerebral artery occlusion. She was not a candidate for intravenous thrombolysis because of a recent cerebral infarction. Attempts at mechanical thrombectomy using a stent retriever, contact aspiration, or combined contact aspiration and stent retriever were unsuccessful. The parallel stent retriever technique using two 6 × 25-mm Trevo stent retrievers enabled coverage of the entire thrombus, and successful reperfusion was achieved (extended Thrombolysis in Cerebral Infarction grade 2b). After the procedure, the patient was able to walk without assistance. Her modified Rankin Scale score was 2 at 90 days follow-up. Microscopic examination of the retrieved thrombi demonstrated red blood cells, fibrin, and partial endothelialization. The parallel Trevo stent retriever technique has the potential as rescue therapy for refractory large-vessel occlusion. However, complications arising from this technique remain uncertain. Further studies are needed to determine the effect of this technique in terms of recanalization and clinical safety.

## 1. Introduction

Despite recent remarkable advances in mechanical thrombectomy (MT) devices in recent years, arterial recanalization [final extended Thrombolysis in Cerebral Infarction grade (eTICI) ≥2b flow] is unsuccessful in ~5%−15% of patients with acute large-vessel occlusion (LVO) who undergo simple stent retriever thrombectomy (SRT) or combined contact aspiration and stent retriever (Liebeskind et al., 2019; LeCouffe et al., 2020; Fischer et al., 2022; Mitchell et al., 2022; Yoshimura et al., 2022). Previous studies have proposed a novel approach using the double SRT as rescue therapy for arterial occlusions that are refractory to first-line SRT. In this technique, the thrombus is trapped between two stents deployed in a Y-configuration (Li et al., 2020; Jiang et al., 2021) or overlapping (Klisch et al., 2015; Asadi et al., 2016; Sasaki et al., 2022) (for one vessel or arterial bifurcations, respectively). After the integration of the thrombus, both stents are pulled out together. Double SRT was reported to be an effective (eTICI from 85% to 100%) and safe procedure (Xu et al., 2021). Therefore, double SRT may become the first-line option in some locations (terminal internal carotid artery or bifurcations) and even for “firm” thrombi.

Although double SRT can capture a large portion of the thrombus at once, there is a high risk of hemorrhagic complications due to the substantial displacement of blood vessels by the two stent retrievers. Most previous reports have been cases where double SRT was performed for intracranial LVO (Klisch et al., 2015; Asadi et al., 2016; Li et al., 2020; Jiang et al., 2021; Xu et al., 2021; Sasaki et al., 2022) or dural sinus thrombosis (Byer et al., 2022). However, when hemorrhagic complications occur in intracranial vessels, the fatal outcome of subarachnoid hemorrhage often follows, and thus, using double SRT as first-line MT is challenging. There have been few reports of parallel SRT (PSRT), in which two Trevo stent retrievers are placed entirely parallel to the extracranial or intracranial-epidural vessels and retrieved as a single unit.

We describe the patient with extracranial and intracranial-epidural internal carotid artery (ICA) occlusion using PSRT to perform rescue thrombectomy with two Trevo stent retrievers (Stryker Neurovascular, Fremont, California, USA) after standard SRT, including a stent retriever, contact aspiration, or combined contact aspiration and stent retriever, had failed.

### 1.1. Case history and clinical examination

**Figure 1:**
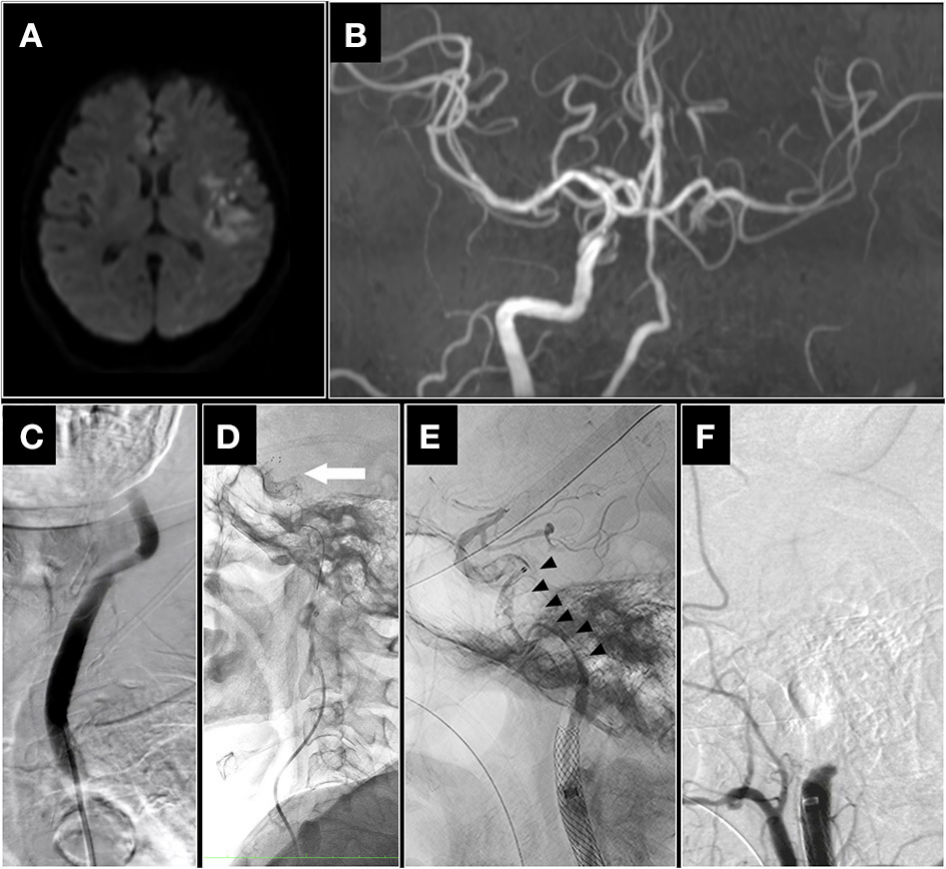
**(A)** Magnetic resonance imaging and angiography findings. Diffusion-weighted imaging revealed acute infarction in the left middle cerebral artery (MCA) territory. **(B)** Magnetic resonance angiography showed occlusion of the left internal carotid artery (ICA). **(C)** Left common carotid artery angiography showed left ICA occlusion (frontal view). **(D)** A 6 × 40-mm Solitaire™ Platinum Revascularization Device (Medtronic, Irvine, California, USA) was deployed from the M1 segment of the MCA to the cavernous portion of the ICA, and a 0.068-inch Penumbra 5MAX ACE aspiration catheter (Penumbra, Alameda, CA, USA) was advanced into the intracranial ICA until the drip rate slowed (arrow; lateral view). **(E)** Intraprocedural left ICA angiography showed a continuous filling defect from the cervical ICA to the intracranial ICA after carotid arterial stenting, which was performed because of iatrogenic ICA dissection after one pass (black arrowhead; lateral view). **(F)** Successful reperfusion was not achieved after standard techniques, as shown by the persisting left ICA occlusion (lateral view).

A 68-year-old woman with a history of hypertension, type 1 diabetes mellitus, dyslipidemia, and ischemic heart disease presented with a disturbance of consciousness and left hemiplegia of abrupt onset, and the baseline National Institutes of Health Stroke Scale score was 17. She underwent MT for acute left middle cerebral artery (MCA) M1 occlusion at our institution, and first-pass eTICI 3 was achieved using SRT. Four days later, she developed disturbed consciousness, mild right hemianopsia, global aphasia, right hemiplegia, and conjugate left eye deviation, and her National Institutes of Health Stroke Scale score was 28. Diffusion-weighted magnetic resonance imaging revealed acute infarction in the left MCA territory (Figure 1A). Magnetic resonance angiography showed a left ICA occlusion, and perfusion of the left MCA was present but reduced, presumably *via* the anterior communicating artery (Figure 1B). The infarct volume was 67 mL, measured by the RAPID automated processing system (iSchemaView, Menlo Park, CA, USA), using an apparent diffusion coefficient index and a time-to-maximum threshold of 620 × 10^−6^ mm^2^/second and >6 s, respectively. She was not a candidate for recombinant intravenous-tissue plasminogen activator because of a recent cerebral infarction; therefore, she underwent MT. Informed consent for the procedure was obtained from the patient's family.

## 2. Endovascular procedure

Left common carotid angiography confirmed a proximal left ICA occlusion. A 0.027-inch microcatheter (Marksman; Stryker, Fremont, CA, USA) was introduced into the 9 Fr balloon guide catheter over a 0.014-inch microguidewire (CHIKAI; ASAHI INTECC, Nagoya, Japan). A 6 × 40 mm Solitaire stent retriever (Medtronic, Irvine, CA, USA) was deployed from the M1 segment of the MCA into the cavernous portion of the ICA. Once the microcatheter was removed, a 0.068-inch Penumbra 5MAX ACE aspiration catheter (Penumbra, Alameda, CA, USA) was advanced to the intracranial ICA until the drip rate slowed (Figure 1C). A 6-mm Solitaire stent retriever (Medtronic, Irvine, California, USA) and a 0.068-inch Penumbra 5MAX ACE aspiration catheter were then retrieved as a single unit into the balloon guide catheter. Successful reperfusion was not achieved, and angiography revealed a large filling defect that extended from the cervical ICA to the intracranial ICA (Figure 1D). After the first pass, angiographic findings showed suspected iatrogenic ICA dissection; thus, we performed carotid artery stenting with 10 × 31-mm Carotid Wallstent RP (Boston Scientific, Marlborough, MA US) for iatrogenic ICA dissection. Successful reperfusion was not achieved after as following technique; combined contact aspiration and stent retriever with a 6-mm Solitaire stent retriever and a 0.068-inch Penumbra 5MAX ACE aspiration catheter; simple SRT with a 6 × 25-mm Trevo stent retriever; and contact aspiration with a 0.068-inch Penumbra 5MAX ACE aspiration catheter (Figure 1E).

### 2.1. Parallel stent retriever thrombectomy

**Figure 2:**
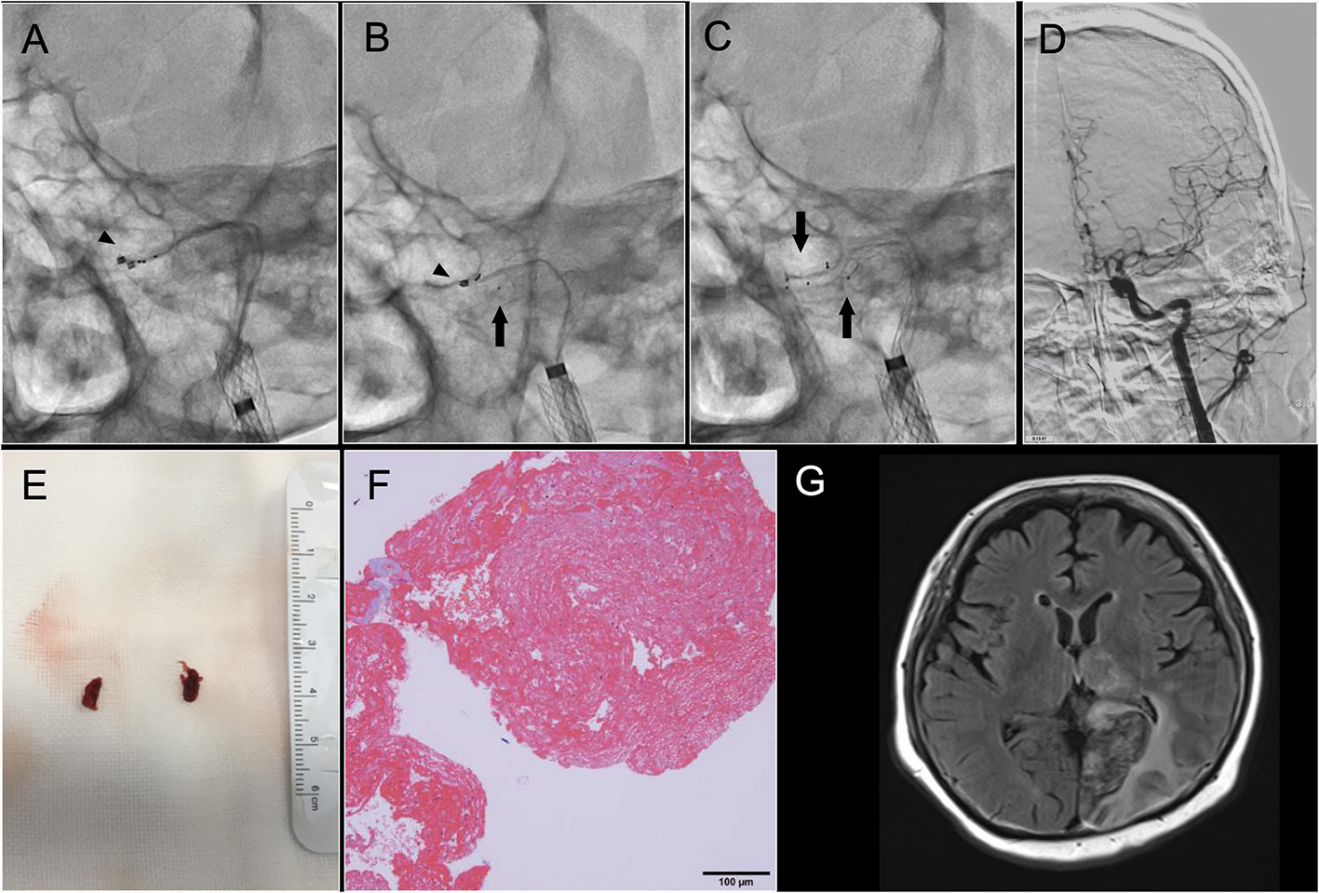
Parallel stent retriever thrombectomy and macroscopic and microscopic findings of the retrieved thrombi. **(A–C)** Two Marksman microcatheters (Stryker, Fremont, CA, USA) were navigated sequentially into the petrous segment of the internal carotid artery (ICA) and placed in parallel with at least one tip distal to the thrombus and ending in the cervical segment of the ICA. Two 6 × 25-mm Trevo™ stent retrievers (Stryker Neurovascular, Fremont, California, USA) were sequentially delivered from each microcatheter to cover the entire thrombus. **(D)** Final angiography showed extended Thrombolysis in Cerebral Infarction grade 2b reperfusion. **(E)** Large red thrombi were retrieved. **(F)** The specimens retrieved by mechanical thrombectomy were histopathologically analyzed. Obtained thrombi were fixed in a phosphate-buffered formalin solution. Formalin-fixed specimens were embedded in paraffin and stained with hematoxylin-eosin. Microscopic examination of the thrombi demonstrated that they were predominantly composed of red blood cells and fibrin and partially endothelialized. **(G)** Magnetic resonance fluid-attenuated inversion recovery imaging 7 days after mechanical thrombectomy. Infarction was observed in the left occipital lobe.

**Figure 3:**
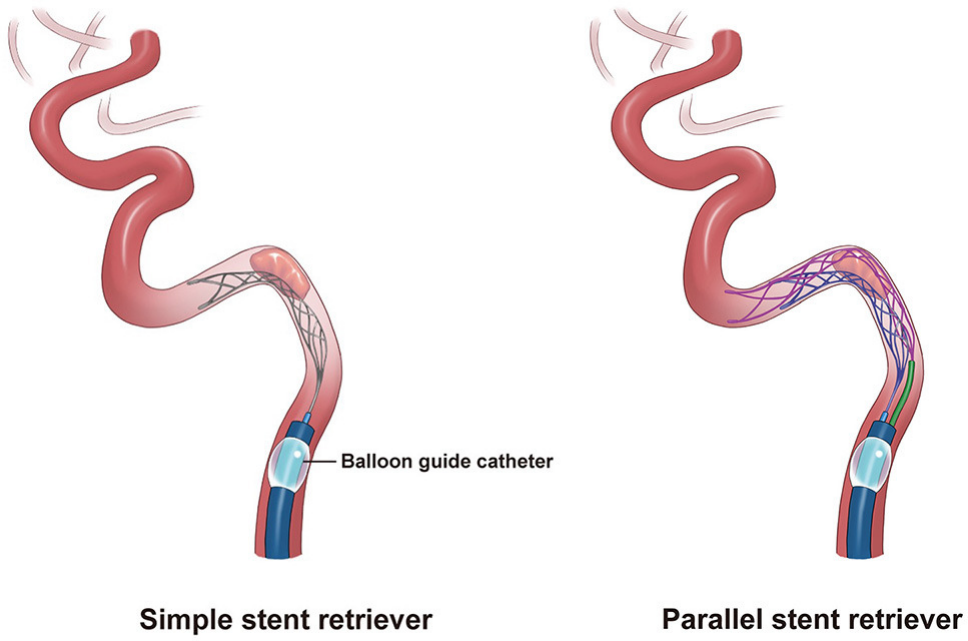
Illustrations of simple and parallel stent retriever thrombectomy. A simple stent retriever deployed adjacent to a large and hard thrombus was unable to capture the thrombus **(left)**. Using the parallel stent retriever technique, the thrombus was captured by interposing the thrombus between two stent retrievers **(right)**.

Two Marksman microcatheters were navigated sequentially into the petrous segment of the ICA (Figure 2A). Both were placed in parallel with at least one tip distal to the thrombus, ending in the cervical segment of the ICA (Figure 2B). A parallel stent retriever configuration enables the retrieval of a large, long, and firm thrombus by increasing the thrombus catchment area (Figure 2C). Then, two 6 × 25-mm Trevo stent retrievers were sequentially delivered from each microcatheter to cover the entire thrombus. Both were slowly recovered by simultaneously pulling them together into the balloon guide catheter under continuous aspiration. Final angiography showed eTICI 2b reperfusion (Figure 2D). The procedure enabled the retrieval of large red thrombi (Figure 2E). Atrial fibrillation was detected on a 24-h electrocardiogram on day 7. The patient was diagnosed with cardioembolism as stroke etiology, and apixaban 10 mg/day was started. After the procedure, non-contrast computed tomography showed no evidence of intracranial hemorrhage, and the patient was able to walk without assistance. The patient has continued aspirin 100 mg and clopidogrel 75 mg once daily for 90 days for secondary prevention, followed by clopidogrel 75 mg once daily, and her modified Rankin Scale score at 90 days was 2. The specimens retrieved by MT were histopathologically analyzed. The obtained thrombi were fixed in a phosphate-buffered formalin solution. Formalin-fixed specimens were embedded in paraffin and stained with hematoxylin-eosin. Microscopic examination of the thrombi demonstrated that they were predominantly composed of red blood cells and fibrin and partially endothelialized (Figure 2F). Magnetic resonance imaging showed cerebral infarction in left occipital lobe 7 days after MT (Figure 2G). Illustrations of the simple SRT and PSRT procedures are shown in Figure 3.

## 3. Discussion

We report the use of PSRT for extracranial or intracranial-epidural ICO using two Trevo stent retrievers after the failure of standard SRT. In our case, the thrombus remained attached to the ICA after thrombectomy attempts using various devices. The stent retriever may not have been sufficiently deployed because the thrombus was large and firm. We attempted aspiration in conjunction with stent retrieval; however, this also failed. The following mechanisms may have enhanced the clot capturing ability of the parallel stent retrievers: firmly capturing the clot between the two stent retrievers strengthened the interaction between the stent retriever and the clot, reduced the frictional force between the clot and the vessel wall, and caused the thrombus to detach from the vessel wall (Figure 3).

Klisch et al. evaluated double SRT in 10 acute intracranial LVO patients (median National Institutes of Health Stroke Scale score of 16; five ICA occlusions and five MCA occlusions) (Asadi et al., 2016), in whom, standard MT had been performed previously in nine patients. They reported one complication and two deaths. The clinical outcome was good in 50% of patients. Although no severe spasms or dissections were reported, mild vasospasm occurred in seven patients.

Despite previous reports that suggest that PSRT is effective as a rescue treatment, concerns surrounding the use of PSRT for intracranial vascular occlusion, which causes more damage to the vascular endothelium than SRT, have not been completely resolved. Simultaneous removal of two stent retrievers may overload the target vessel, and if significant resistance is encountered when recovering the retrievers, the removal of each one individually may be required to avoid vessel injury. In addition, PSRT is complicated and is recommended only as a rescue technique because simple SRT achieves favorable outcomes in 70%−80% of cases (Nogueira et al., 2012; Campbell et al., 2015). Although PSRT has previously been performed in patients with LVO, caution must be exercised because of the risk of vascular injury and subsequent intracranial hemorrhage. Depending on the positional relationship between the stent retrievers and the thrombus, PSRT can result in a strong force being applied to the vessel. Vascular traction is milder when the thrombus is captured using stent retrievers with aspiration assistance. Given the balance between the thrombus-captured force and the minimal damage to blood vessels, there may be less vascular shift and minimal vascular damage when parallel stent retrievers are deployed relatively close to the balloon guide or aspiration catheter (Supplementary Video 1). Hernández et al. compared the degree of vascular injury between simple SRT and PSRT in 36 samples. Compared with simple SRT, PSRT showed greater histological damage, corresponding to the damage caused by 1.4 times simple SRT passes (Hernández et al., 2022). Considering the risk of vascular injury, PSRT may be more appropriate and safer to perform in patients with intracranial-epidural LVO, as demonstrated in the present case.

## 4. Conclusion

We reported a patient with an ICA occlusion who underwent successful MT using PSRT with two Trevo stent retrievers as rescue therapy after other techniques had failed. The parallel SR technique has the potential as rescue therapy for refractory LVO. Further studies are needed to determine the effect of this technique in terms of recanalization and clinical safety and characterize the risk-benefit ratio of PSRT in routine clinical practice.

## Data availability statement

The original contributions presented in the study are included in the article/Supplementary material, further inquiries can be directed to the corresponding author.

## Ethics statement

The studies involving human participants were reviewed and approved by National Cerebral and Cardiovascular Center. The patients/participants provided their written informed consent to participate in this study. Written informed consent was obtained from the individual(s) for the publication of any potentially identifiable images or data included in this article.

## Author contributions

TY collected the data and wrote the manuscript. SH, KTa, and JK collected the data and revised the manuscript. TS, HY, KTo, and MI supervised the writing and revising. All authors contributed to and approved the final version of the manuscript.

## Supplementary material

Supplementary Video 1 Mechanical thrombectomy using the parallel stent retriever technique.

## References

[B1] Asadi H. Brennan P. Martin A. Looby S. O'Hare A. Thornton J.. (2016). Double stent retriever technique in endovascular treatment of middle cerebral artery saddle embolus. J. Stroke Cerebrovasc. Dis. 25, e9–11. 10.1016/j.jstrokecerebrovasdis.2015.10.005 26698640

[B2] Byer S. H. Madarang E. J. Abraham M. G. (2022). Dual-Stent retriever thrombectomy for extensive dural sinus thrombosis. Int. J. Neurosci. 13, 1–6. 10.1080/00207454.2022.2080675 35593753 PMC9744960

[B3] Campbell B. C. Mitchell P. J. Kleinig T. J. Dewey H. M. Churilov L. Yassi N.. (2015). Endovascular therapy for ischemic stroke with perfusion-imaging selection. N Engl. J. Med. 372, 1009–1018. 10.1056/NEJMoa1414792 25671797

[B4] Fischer U. Kaesmacher J. Strbian D. Eker O. Cognard C. Plattner P. S.. (2022). Thrombectomy alone versus intravenous alteplase plus thrombectomy in patients with stroke: an open-label, blinded-outcome, randomised non-inferiority trial. Lancet 400, 104–115. 10.1016/S0140-6736(22)00537-2 35810756

[B5] Hernández D Cuevas J. L. Gramegna L. L. Requena M. Piñana C. de Dios M Increased number of passes double stent retriever technique induces cumulative injury on arterial wall after mechanical thrombectomy in a swine model. Transl. Stroke Res. (2022) 2022 1–9. 10.1007/s12975-022-01054-z. 35672562

[B6] Jiang C. Li Y. Hao F. Yang J. Wang B. Fan Y.. (2021). Y-configuration double-stent retriever thrombectomy for refractory thrombus in middle cerebral artery bifurcation: a case report. Medicine (Baltimore) 100, e24993. 10.1097/MD.0000000000024993 33725971 PMC7982166

[B7] Klisch J. Sychra V. Strasilla C. Taschner C. A. Reinhard M. Urbach H.. (2015). Double solitaire mechanical thrombectomy in acute stroke: effective rescue strategy for refractory artery occlusions? Am. J. Neuroradiol. 36, 552–556. 10.3174/ajnr.A4133 25324495 PMC8013066

[B8] LeCouffe N. E. Kappelhof M. Treurniet K. M. Lingsma H. F. Zhang G. van den Wijngaard I. R.. (2020). 2B, 2C, or 3: what should be the angiographic target for endovascular treatment in ischemic stroke? Stroke. 51, 1790–1796. 10.1161/STROKEAHA.119.028891 32397926

[B9] Li Z. Liu P. Zhang L. Zhang Y. Fang Y. Xing P.. (2020). Y-stent rescue technique for failed thrombectomy in patients with large vessel occlusion: a case series and pooled analysis. Front. Neurol. 11, 924. 10.3389/fneur.2020.00924 32973671 PMC7481477

[B10] Liebeskind D. S. Bracard S. Guillemin F. Jahan R. Jovin T. G. Majoie C. B.. (2019). eTICI reperfusion: defining success in endovascular stroke therapy. J. Neurointerv. Surg. 11, 433–438. 10.1136/neurintsurg-2018-014127 30194109

[B11] Mitchell P. J. Yan B. Churilov L. Dowling R. J. Bush S. J. Bivard A.. (2022). Endovascular thrombectomy versus standard bridging thrombolytic with endovascular thrombectomy within 4.5 H of stroke onset: an open-label, blinded-endpoint, randomised non-inferiority trial. Lancet 400, 116–125. 10.1016/S0140-6736(22)00564-5 35810757

[B12] Nogueira R. G. Lutsep H. L. Gupta R. Jovin T. G. Albers G. W. Walker G. A.. (2012). TREVO 2 trialists. trevo versus merci retrievers for thrombectomy revascularisation of large vessel occlusions in acute ischaemic stroke (TREVO 2): a randomized trial. Lancet 380, 1231–1240. 10.1016/S0140-6736(12)61299-9 22932714 PMC4176618

[B13] Sasaki I. Imahori T. Yano T. Gomi M. Kuroda J. Kobayashi N.. (2022). Crossing double stent retriever technique for refractory terminal internal carotid artery occlusion. Radiol. Case Rep. 17, 1848–1852. 10.1016/j.radcr.2022.03.023 35401893 PMC8990047

[B14] Xu H. Peng S. Quan T. Yuan Y. Wang Z. Fu X.. (2021). Tandem stents thrombectomy as a rescue treatment for refractory large vessel occlusions. J. Neurointerv. Surg. 13, 33–38. 10.1136/neurintsurg-2020-015822 32641417

[B15] Yoshimura Y. Sakai N. Yamagami H. Uchida K. Beppu M. Toyoda K.. (2022). Endovascular therapy for acute stroke with a large ischemic region. N. Engl. J. Med. 386, 1303–1313. 10.1056/NEJMoa2118191 35138767

